# Impact of environmental factors on marijuana use in 11 European countries

**DOI:** 10.3325/cmj.2011.52.446

**Published:** 2011-08

**Authors:** Iva Pejnović Franelić, Marina Kuzman, Ivana Pavić Šimetin, Josipa Kern

**Affiliations:** 1 School and Adolescent Medicine and Addiction Prevention Service, Croatian National Institute of Public Health, Zagreb, Croatia; 2 Department of Medical Statistics, Epidemiology and Medical Informatics Andrija Štampar School of Public Health, School of Medicine, University of Zagreb, Zagreb, Croatia

## Abstract

**Aim:**

To investigate the association between environmental factors (perceived availability of marijuana, perceived use among friends and siblings, use of alcohol and tobacco, family structure, parental control, school performance) and lifetime prevalence and frequent and early marijuana use in high school students.

**Methods:**

We used self-reported data from 15-16 years old participants of the 2003 European School Survey Project on Alcohol and Other Drugs (ESPAD) conducted in 11 countries: Denmark, Estonia, Norway, Croatia, Slovenia, Germany, Switzerland, Bulgaria, Czech Republic, Russian Federation, and Ukraine. Multivariate logistic regression was used for data analysis.

**Results:**

Countries varied according to lifetime prevalence (8.7%-47.8%) and frequent (8.7%-23.9%) and early (3.0%-13.0%) marijuana use. Daily tobacco smoking was most strongly associated with lifetime marijuana use for boys in 7 and for girls in 5 countries, with highest odds ratio (OR, 95% and confidence interval – CI) for boys in Denmark (OR, 13.52; 95% CI, 8.16-22.4), and for girls in the Czech Republic (OR, 21.21; 95% CI, 12.99-34.62). Perceived marijuana availability was most strongly associated with frequent marijuana use for boys in 4 countries (highest in Slovenia: OR, 19.28; 95% CI, 6.52-57.02) and girls in 5 (highest in Slovenia: OR, 19.05; 95% CI, 5.18-70.04). Perceived use of marijuana among friends was most strongly associated with frequent marijuana use in 5 countries, both for boys (highest in Norway: OR, 23.91; 95% CI, 4.16-137.48) and girls (highest in Denmark: OR, 75.42; 95% CI, 13.11-433.90). Perceived use of marijuana among friends was most strongly associated with early marijuana use in 8 countries for boys (highest in Norway: OR, 54.03; 95% CI, 3.34-875.19) and 3 countries for girls (highest in Denmark: OR, 7.29; 95%CI, 1.77-30.12).

**Conclusion:**

In each country, marijuana use was associated with similar factors, regardless of marijuana use prevalence in that country.The influence of peer group and perceived availability of marijuana seemed more important than parental control and family structure.

Following tobacco and alcohol, cannabis continues to be the psychoactive substance most commonly used by school children (1). Epidemiological research during the past 10 years suggests that regular use of cannabis during adolescence and into adulthood can have adverse effects. The most probable adverse effects include a dependence syndrome, increased risk of motor vehicle accidents, impaired respiratory function, risk of cardiovascular disease, and adverse effects on adolescents’ psychosocial development and mental health (2). There is substantial evidence that alcohol, tobacco, and cannabis dependence problems surface more quickly when use of these drugs starts before adulthood (3). Early and regular cannabis use in adolescence predicts an increased risk of cannabis dependence, which in turn predicts an increased risk of using other illicit drugs (4). Frequent cannabis use in late adolescence and early adulthood is associated with a range of adverse outcomes in later life (5). Adolescent substance use is directly affected by peer influence (6), while parent-family connectedness is protective for health risk behavior (7). It was found that authoritative parenting style leads to better adolescent school performance and stronger school engagement (8), while parental monitoring, open parent-child communication, supervision, and high quality of the parent-child relationship deter involvement in high-risk behavior (9). Both parents and peers can have strong influences on adolescents, depending on the arena of influence. Parents are particularly important for future life plans, while peers are most important for involvement in illicit drug use (10). However, for drug use itself, there are different patterns of influence depending upon the stage of drug involvement. Peers are especially important for initiation into marijuana use, while parental factors gain in importance in the transition from marijuana use to the use of other illicit drugs (10). The 2003 European School Survey Project on Alcohol and Other Drugs (ESPAD) Report by Hibell et al found that association between adolescent substance use and family background was complex and dependent upon the type of substance, element of family background, and the country of study (11). Generally, the strongest correlates of substance use by adolescent students were going out most evenings, substance use by peers and siblings, and antisocial behavior (12).

In this study, we investigated the influence of contextual factors on lifetime marijuana use, frequent marijuana use (10 times or more in the lifetime), and early onset of marijuana use (13 years or younger) by sex in 11 European countries.

## Methods

The data used in this study were obtained from the cross-sectional school population ESPAD survey, following standardized methodology carried out in 2003 (11). The main purpose of the ESPAD project was to collect comparable data on alcohol, tobacco, and drug use among 15-16 years old students in European countries and to compare the trends between countries and between groups of countries. The study was conducted as school surveys by researchers in each country, during the same period and with common methodology (11).

Eleven countries (Denmark, Estonia, Norway, Croatia, Slovenia, Germany, Switzerland, Bulgaria, the Czech Republic, Russian Federation – Moscow, and Ukraine), whose data were available and eligible for the current study cover all European geographic regions (13). From each region, at least two countries were available. According to the ESPAD study sampling procedure, a national probability sample of 15-16 years old students was drawn for each country (11).

### Instrument

Anonymous questionnaires were administered to all the students born in 1987 who were present in class on the day of the survey in spring 2003. The data collection in a country was planned to take place during a week that was not preceded by any holiday, ensuring that the students referred to a “normal” week when answering the questions. It means that no extraordinary alcohol or drug consumption due to any celebration was reflected in answers. Data were collected by group-administered questionnaires in schools on nationally representative samples of classes. Exceptions include Russian Federation, where the study was restricted to Moscow only and Germany, where the study was performed in six federal states. The students themselves sealed questionnaires in unmarked envelopes, which were sent to the research institution. The complete questionnaire is given in English in Hibell et al (11).

### Measurements

Lifetime prevalence of marijuana use and frequent marijuana use were assessed by asking pupils on how many occasions (if any) they had used marijuana (grass, pot) or hashish (hash, hash oil) in their lifetime with response options ranging from “0” to “40 or more.” Lifetime prevalence of marijuana use was defined as use of marijuana on at least one occasion in life. Frequent marijuana use was defined as use of marijuana on 10 or more occasions in life. Early initiation of marijuana use was assessed by asking pupils when (if ever) they first tried marijuana or hashish with the following response options: 11 years old or less, 12, 13, 14, 15, 16 years old, and never. Early initiation of marijuana use was defined as trying marijuana for the first time at the age 13 or younger.

Parental control was assessed by asking pupils weather their parents knew where they spent Saturday nights with four response options ranging from “know always” to “usually don’t know.”Answers “usually don’t know” were compared to all other answers. Family structure was assessed by asking pupils who lived in the same household with them. Living with father and mother (with or without other family members) was defined as intact family structure and compared with living with one parent or without parents. Perceived availability of marijuana was assessed by asking pupils how difficult it would be for them to get marijuana or hashish if they wanted. There were six response options, ranging from “impossible” to “very easy,” with “don’t know” as one of the options. Responses “fairly easy” and “very easy” were compared to other responses. Marijuana use among friends was assessed by asking pupils to estimate how many of their friends smoked marijuana (pot, grass) or hashish. Response options ranged from “none” to “all.” Those who answered “most” and “all” were compared to those who answered fewer than most. Marijuana use among older siblings was assessed by asking pupils if any of their older siblings smoked marijuana or hashish (pot, grass) with response options: “yes,” “no,” “don’t know,” and “don’t have any older siblings.” Positive answers were compared to negative and indecisive answers (those who don’t have any older siblings were left out from the analyses). Alcohol use was assessed by asking pupils on how many occasions (if any) they drank any alcoholic beverage during the last 30 days. Response options were 0, 1-2, 3-5, 6-9, 10-19, 20-39, and 40 or more. Drinking on 6 or more occasions (labeled as frequent alcohol use) was compared to less frequent drinking. Smoking cigarettes was assessed by asking pupils how frequently they smoked cigarettes during the last 30 days. Response options were not at all, fewer than 1 cigarette per week, fewer than 1 cigarette per day, 1-5 cigarettes per day, 6-10 cigarettes per day, 11-20 cigarettes per day, and more than 20 cigarettes per day. Smoking at least one cigarette daily (daily smoking) was compared to less frequent smoking. School performance was assessed by asking pupils which response category described best their average grade at the end of the last term. Two highest (very good/excellent) out of five possible response categories were compared to less successful school performance.

### Variables

Three dependent variables were used in the analysis: lifetime prevalence of marijuana use (yes, no), marijuana use 10 times or more in lifetime (yes, no), and marijuana use at the age of 13 or earlier (yes, no).

Eight independent variables were daily smoking (at least one cigarette a day in the last month = yes, otherwise = no), frequent alcohol use (6 times or more in the last month = yes, otherwise = no), perceived marijuana availability (easy/fairly or easy = yes, otherwise = no), perception of having friends who use marijuana (most or all = yes, otherwise = no), perception of having older siblings who use marijuana sometimes (yes, no), parental control (parents usually do not know where students spend Friday/Saturday night = yes, otherwise = no), school performance (very good/excellent = yes, otherwise = no), and family structure (living with both parents = yes, otherwise = no).

### Statistical analysis

Multivariate logistic regression model was applied to lifetime prevalence of marijuana use, frequent marijuana use, and early initiation of marijuana use as dependent variables and eight binary environmental variables as potential influencing factors separately by sex. Odds ratios (OR) with 95% confidence intervals (95% CI) and level of significance (*P*) were calculated. All the analyses were carried out by using SAS (SAS Institute Inc., Cary, NC, USA) statistical software package.

## Results

Total sample from 11 countries was 34 193 respondents (16 521 men and 17 672 women) from Denmark (n = 2519), Estonia (n = 2463), Norway (n = 3833), Croatia (n = 2884), Slovenia (n = 2785), Germany (n = 5087), Switzerland (n = 2613), Bulgaria (n = 2739), the Czech Republic (n = 3172), Russian Federation (Moscow) (n = 1925), and Ukraine (n = 4173) (Tables 1 and 2).

**Table 1:** Environmental factors or behaviors associated with marijuana use in male (M) and female (F) participants aged 15-16 years*

			Environmental factor or behavior, n (% within the country)					
Country	Sex	Total number	marijuana use at age 13 or younger	lifetime marijuana use	marijuana use >10 times in life	daily tobacco smoking the country)	frequent alcohol use	marijuana available “very easy” or “fairly easy”
Denmark	M	1248	79 (6.4)	332 (26.9)	109 (8.8)	232 (18.6)	**391 (32.5)**	660 (53.3)
	F	1271	59 (4.7)	233 (18.4)	62 (4.9)	255 (20.1)	273 (22.5)	628 (49.6)
Estonia	M	1246	79 (6.5)	345 (27.9)	112 (9.1)	388 (31.2)	193 (15.9)	320 (26.1)
	F	1217	29 (2.4)	217 (18.0)	36 (3.0)	277 (22.9)	145 (12.3)	242 (20.1)
Norway	M	1945	**56 (3.0)**	**164 (8.7)**	**54 (2.9)**	**296 (15.4)**	145(8.3)	476 (25.1)
	F	1888	39 (2.1)	**168 (9.1)**	31 (1.7)	399 (21.3)	**118(6.9)**	495 (26.8))
Croatia	M	1446	63 (4.4)	346 (24.0)	129 (8.9)	411 (28.4)	414 (29.0)	624 (43.5)
	F	1438	45 (3.1)	293 (20.4)	92 (6.4)	392 (31.6)	219 (15.4)	660 (46.2)
Slovenia	M	1406	116 (8.3)	429 (30.7)	171 (12.2)	316 (22.5)	255 (19.0)	782 (56.5)
	F	1379	81 (5.9)	360 (26.1)	131 (9.5)	346 (25.1)	120 (9.0)	730 (53.3)
Germany	M	2402	234 (9.8)	781 (32.6)	330 (13.8)	807 (33.7)	653 (27.6)	1072 (45.1)
	F	2685	245 (9.1)	674 (25.2)	219 (8.2)	1003 (37.4)	559 (21.3)	1056 (39.6)
Switzerland	M	1278	**161 (13.0)**	563 (44.2)	**305 (23.9)**	274 (21.5)	387 (30.4)	698 (55.1)
	F	1335	**121 (9.2)**	477 (35.8)	**207 (15.5)**	277 (20.8)	225 (16.9)	619 (46.9)
Bulgaria	M	1290	49 (3.9)	293 (23.2)	108 (8.6)	425 (33.4)	286 (23.4)	437 (34.9)
	F	1449	35 (2.4)	271 (18.8)	75 (5.2)	**559 (38.7)**	210 (15.1)	1056 (37.3)
Czech Republic	M	1462	92 (6.3)	**696 (47.8)**	305 (21.0)	423 (29.1)	457 (31.7)	**867 (60.0)**
	F	1710	111 (6.5)	**680 (40.0)**	257 (15.1)	432 (25.3)	345 (20.4)	**948 (55.8)**
Russian Federation (Moscow)	M	880	44 (5.1)	225 (25.7)	53 (6.1)	**324 (36.9)**	217 (25.8)	213 (24.6)
	F	1045	28 (2.7)	192 (18.5)	34 (3.3)	329 (31.6)	196 (19.3)	238 (23.0)
Ukraine	M	1918	75 (4.0)	515 (27.3)	125 (6.6)	629 (32.8)	251 (13.8)	**316 (16.9)**
	F	2255	**22 (1.0)**	251 (11.2)	**29 (1.3)**	**326 (14.5)**	196 (9.2)	**184 (8.4)**

*The highest and the lowest values for each factor by sex are in bold.

**Table 2:** Environmental factors or behaviors associated with marijuana use in male (M) and female (F) aged 15-16 years*

			Environmental factors or behaviors, n (% within the country)				
Country	Sex	Total number	most or all friends smoke marijuana	older siblings smoke marijuana	parents usually do not know where pupils spend Saturday evenings	school performance very good/excellent	living with both parents
Denmark	M	1248	50 (4.0)	129 (14.3)	**21 (1.7)**	279 (22.4)	906 (73.0)
	F	1271	27 (2.1)	136 (15.5)	12 (0.9)	421 (33.3)	893 (70.6)
Estonia	M	1246	80 (6.6)	69 (7.2)	**106 (8.7)**	207 (16.8)	**783 (63.6)**
	F	1217	53 (4.4)	49 (5.8)	70 (5.8)	383 (31.5)	**715 (59.0)**
Norway	M	1945	**32 (1.7)**	97 (7.7)	88 (4.7)	316 (17.2)	1357 (71.8)
	F	1888	**31 (1.7)**	95 (7.8)	**12 (0.6)**	560 (31.2)	1341 (72.5)
Croatia	M	1446	117 (8.2)	89 (8.1)	81 (5.7)	**828 (57.4)**	**1167 (82.5)**
	F	1438	123 (8.6)	85 (8.2)	57 (4.0)	**1091 (76.0)**	1164 (81.3)
Slovenia	M	1406	138 (10.0)	78 (7.0)	76 (5.5)	**73 (5.2)**	1122 (80.1)
	F	1379	144 (10.6)	102 (9.9)	51 (3.7)	**122 (8.9)**	**1134 (82.5)**
Germany	M	2402	200 (8.4)	143 (10.4)	96 (4.0)	839 (35.1)	1766 (74.1)
	F	2685	164 (6.2)	192 (12.6)	61 (2.3)	1245 (46.5)	1914 (71.7)
Switzerland	M	1278	191 (15.1)	148 (19.1)	45 (3.6)	687 (54.1)	983 (78.2)
	F	1335	**204 (15.5)**	162 (20.9)	31 (2.3)	850 (64.1)	1021 (77.1)
Bulgaria	M	1290	89 (7.2)	58 (6.6)	**111 (8.7)**	331 (25.8)	965 (77.5)
	F	1449	102 (7.2)	66 (7.1)	**97 (6.8)**	683 (47.4)	1099 (76.9)
Czech Republic	M	1462	**188 (13.1)**	**199 (20.2)**	60 (4.2)	444 (31.0)	1039 (72.7)
	F	1710	230 (13.6)	**248 (22.6)**	54 (3.2)	838 (50.7)	1213 (71.4)
Russian Federation (Moscow)	M	880	46 (5.3)	42 (6.4)	65 (7.5)	102 (11.7)	550 (63.8)
	F	1045	53 (5.1)	72 (9.9)	49 (4.7)	211 (20.2)	670 (64.4)
Ukraine	M	1918	105 (5.6)	**85 (6.0)**	98 (5.1)	305 (16.1)	1217 (64.3)
	F	2255	42 (1.9)	**83 (5.0)**	59 (2.6)	748 (33.6)	1475 (65.8)

*The highest and the lowest values for each factor by sex are in bold.

At the time of data collection, the average age of respondents was 15.8 years. Response rates in 11 countries ranged between 80 and 95%.

Comparison between 11 countries showed a wide variation of lifetime prevalence of marijuana use, frequent marijuana use, and early initiation of marijuana use (Table 1). In all analyzed countries (except Norway), boys used marijuana more frequently (Table 1). The age of first use was lower in the high-prevalence countries (with the exception of the Czech Republic). In 6 out of 11 countries, girls smoked more than boys (Denmark, Norway, Germany, Bulgaria, Croatia, and Slovenia) (Table 1), while in all countries boys drank more than girls (Table 1). Marijuana availability was perceived to be very high (more than 40% respondents of both sexes) in Denmark, Croatia, Slovenia, Switzerland, and the Czech Republic (Table 1).

Prevalence of perceived marijuana use among friends ranged from 1.7% to 15.1% among boys and 1.7% to 15.5% among girls (Table 2), being higher in Switzerland, the Czech Republic, Slovenia, Croatia, and Germany. In the majority of countries, perceived use of marijuana among older siblings was higher than the perceived use of marijuana among friends (Table 2). The number of parents who usually did not know where their children spent Friday/Saturday nights ranged from 1.7% to 8.7% for boys and from 0.6% to 6.8% for girls (Table 2). Very good/excellent school performance was recorded in 5.2% to 57.4% of boys and 8.9% to 76.0% of girls (Table 2). The number of children not living with both biological parents ranged from 36.4% to 17.5% for boys and from 41% to 17.5% for girls (Table 2).

Among boys in all 11 countries, higher odds ratios for marijuana use at least once in lifetime were associated with daily tobacco smoking, perceived high marijuana availability, perceived marijuana use among friends, and perceived marijuana use among older siblings (Table 3 and 4). In all countries except Norway the same was true for frequent alcohol use (Table 3).

**Table 3:** Multivariate logistic regression analysis of lifetime prevalence of marijuana use by sex*

Country	Sex	Marijuana available “very easy” or “fairly easy”	Most or all friends smoke marijuana	Daily smoking	Frequent alcohol use
Denmark	M	5.58 (3.39-9.18)	9.26 (3.03-28.26)	**13.52 (8.16-22.4)**	2.33 (1.52-3.56)
	F	3.48 (2.01-6.03)	**17.28 (3.14-95.11)**	11.30 (6.82-18.73)	2.07 (1.27-3.37)
Estonia	M	5.02 (3.42-7.38)	**10.35 (4.19-25.56)**	3.62 (2.48-5.28)	1.91 (1.20-3.01)
	F	3.91 (2.42-6.32)	4.59 (1.85-11.40)	**6.59 (4.04-10.74)**	2.88 (1.64-5.04)
Norway	M	4.89 (2.80-8.56)	**36.94 (6.65-205.31)**	9.63 (5.40-17.16)	1.64 (0.79-3.41)
	F	6.20 (3.61-10.64)	**12.53 (2.18-72.14)**	5.23 (2.99-9.13)	2.36 (1.18-4.72)
Croatia	M	3.70 (2.54-5.39)	2.27 (1.24-4.16)	**9.78 (6.71-14.24)**	1.49 (1.02-2.19)
	F	6.37 (4.00-10.14)	4.11 (2.28-7.40)	**9.01 (5.92-13.67)**	2.02 (1.24-3.29)
Slovenia	M	5.62 (3.81-8.26)	2.34 (1.32-4.16)	**8.62 (5.74-12.94)**	2.22 (1.47-3.35)
	F	6.58 (4.21-10.28)	4.57 (2.45-8.39)	**9.25 (6.14-13.93)**	1.86 (1.00-3.48)
Germany	M	**8.22 (5.98-11.28)**	3.43 (1.90-6.19)	3.94 (2.85-5.43)	1.52 (1.10-2.10)
	F	**9.76 (6.87-13.85)**	4.45 (2.35-8.44)	7.43 (6.24-10.54)	1.91 (1.34-2.71)
Switzerland	M	6.05 (4.07-8.99)	2.46 (1.35-4.45)	**7.52 (4.33-13.08)**	2.17 (1.44-3.26)
	F	**10.03 (6.36-15.83)**	2.71 (1.54-4.77)	8.30 (4.90-14.06)	2.40 (1.38-4.20)
Bulgaria	M	4.97 (3.25-7.60)	3.97 (1.80-8.75)	**5.91 (3.83-9.11)**	1.83 (1.17-2.87)
	F	6.45 (4.00-10.49)	3.28 (1.61-6.67)	**8.66 (5.09-14.72)**	1.49 (0.87-2.57)
Czech Republic	M	3.07 (2.19-4.3)	3.56 (1.92-6.59)	**5.81 (3.89-8.70)**	2.17 (1.52-3.11)
	F	3.69 (2.51-5.40)	3.06 (1.63-5.74)	**21.21 (12.99-34.62)**	2.52 (1.61-3.969)
Russian Federation (Moscow)	M	3.42 (2.07-5.65)	3.21 (1.27-8.11)	**6.07 (3.83-9.63)**	1.68 (1.04-2.74)
	F	3.70 (2.19-6.26)	**7.49 (2.52-22.24)**	6.26 (3.68-10.66)	1.90 (1.09-3.33)
Ukraine	M	**5.33 (3.67-7.75)**	2.89 (1.47-5.66)	5.01 (3.70-6.80)	1.50 (1.00-2.23)
	F	3.64 (2.19-6.04)	**7.37 (2.32-23.39)**	7.24 (4.87-10.78)	3.50 (2.16-5.66)

*M – male; F – female. The highest odds ratios for a respective country by sex in bold. Results are presented as odds ratios and 95% confidence intervals.

**Table 4:** . Multivariate logistic regression analysis of lifetime prevalence of marijuana use by sex*

Country	Sex	Older siblings smoke marijuana	Parents usually do not know where pupils spend Saturday evenings	Not living with both parents	School performance very good/excellent
Denmark	M	3.61 (2.09-6.24)	1.05 (0.25-4.41)	0.93 (0.57-1.51)	1.52 (0.88-2.63)
	F	6.96 (4.00-12.12)	9.64 (1.35-68.63)	1.83 (1.14-2.03)	1.60 (0.89-2.88)
Estonia	M	2.64 (1.32-5.30)	1.30 (0.70-2.40)	0.92 (0.63-1.34)	1.54 (0.86-2.74)
	F	2.90 (1.28-6.53)	1.23 (0.58-2.63)	0.98 (0.62-1.54)	1.10 (0.64-1.91)
Norway	M	5.79 (2.81-11.95)	0.58 (0.19-1.79)	0.92 (0.50-1.68)	1.46 (0.52-4.13)
	F	3.67 (1.89-7.12)	1.84 (0.61-5.59)	1.68 (0.99-2.85)	1.21 (0.62-2.37)
Croatia	M	3.97 (2.18-7.22)	1.23 (0.62-2.63)	1.01 (0.61-1.65)	1.23 (0.85-1.78)
	F	3.06 (1.68-5.56)	0.85 (0.36-2.02)	1.11 (0.66-1.86)	1.52 (0.98-2.34)
Slovenia	M	4.04 (1.95-8.39)	4.02 (1.97-8.20)	1.06 (0.68-1.64)	1.60 (0.62-4.09)
	F	2.39 (1.33-4.30)	1.56 (0.68-3.56)	1.60 (0.96-2.66)	1.20 (0.57-2.50)
Germany	M	4.90 (2.76-8.70)	1.79 (0.83-3.87)	1.20 (0.85-1.71)	1.12 (0.79-1.57)
	F	3.84 (2.49-5.93)	0.65 (0.28-1.51)	1.08 (0.75-1.54)	1.27 (0.91-1.79)
Switzerland	M	3.43 (2.06-5.72)	1.52 (0.53-4.32)	1.08 (0.67-1.76)	1.39 (0.94-2.04)
	F	2.47 (1.52-3.99)	0.85 (0.22-3.34)	1.19 (0.73-1.94)	1.19 (0.78-1.82)
Bulgaria	M	4.14 (1.80-9.50)	1.19 (0.61-2.24)	1.01 (0.61-1.66)	0.96 (0.58-1.60)
	F	1.95 (0.97-3.97)	0.81 (0.38-1.71)	2.21 (1.31-3.72)	1.04 (0.63-1.71)
Czech Republic	M	2.80 (1.84-4.3)	1.59 (0.65-3.88)	1.46 (1.00-2.13)	1.33 (0.94-1.89)
	F	3.85 (2.48-5.96)	0.80 (0.23-2.80)	1.32 (0.89-1.04)	1.32 (0.92-1.89)
Russian Federation (Moscow)	M	4.71 (2.10-10.56)	0.85 (0.37-1.92)	1.36 (0.86-2.14)	0.94 (0.47-1.89)
	F	4.01 (1.91-8.44)	1.67 (0.66-4.20)	1.37 (0.82-2.30)	1.16 (0.60-2.25)
Ukraine	M	2.75 (1.51-5.02)	0.76 (0.40-1.44)	1.12 (0.82-1.52)	1.96 (1.20-3.21)
	F	5.14 (2.70-9.78)	1.81 (0.70-4.73)	1.32 (0.90-1.95)	1.28 (0.81-2.02)

*M – male; F – female. Results are presented as odds ratios and 95% confidence intervals.

Lifetime prevalence of marijuana use among girls was associated with daily smoking and perceived marijuana use among friends in all countries. Perceived marijuana availability was associated with lifetime marijuana prevalence in all countries except Ukraine and perceived use by siblings in all countries except Bulgaria, and frequent alcohol use in all countries except Slovenia and Bulgaria (Table 3 and 4).

In seven out of eleven countries (Denmark, Croatia, Slovenia, Switzerland, Bulgaria, Czech Republic and Russian Federation), the strongest associated factor for boys was daily smoking.

For two countries (Estonia and Norway), the strongest associated factor was perceived marijuana use among friends and for two countries (Germany and Ukraine) it was perceived marijuana availability (Table 3).

In five out of eleven countries (Estonia, Croatia, Slovenia, Bulgaria, and the Czech Republic), the strongest associated factor for girls was daily smoking.

In four countries (Denmark, Norway, Russian Federation and Ukraine), the strongest associated factor was perceived marijuana use among friends and in two countries (Germany and Switzerland), it was perceived marijuana availability (Table 3).

The same contextual factors that were associated with higher odds ratios for lifetime marijuana use among boys in all countries (ie, perceived marijuana availability, perceived marijuana smoking among friends, and daily smoking) were associated with higher odds ratios for frequent marijuana use among boys in all countries as well (Table 5).

**Table 5:** Multivariate logistic regression analysis of frequent marijuana use (10+ times) by sex*

Country	Sex	Marijuana available “very easy” or “fairly easy”	Most or all friends smoke marijuana	Daily smoking	Frequent alcohol use
Denmark	M	16.08 (3.69-70.07)	**16.88 (6.37-44.74)**	12.34 (6,25-24.36)	0.91 (0.48-1.73)
	F	4.90 (1.07-22.53)	**75.42 (13.11-433.90)**	15.45 (5.41-44.14)	3.75 (1.64-8.57)
Estonia	M	5.37 (2.85-10.12)	**12.32 (5.92-25.67)**	3.78 (2.01-7.12)	1.62 (0.84-3.11)
	F	6.63 (1.95-22.55)	**10.88 (3.38-35.01)**	4.13 (1.17-14.63)	3.53 (1.14-10.93)
Norway	M	16.13 (3.51-744.04)	**23.91 (4.16-137.48)**	21.03 (5.66-78.15)	3.91 (1.43-10.64)
	F	**16.00 (1.94-131.80)**	4.73 (0.84-26.65)	2.74 (0.64-11.64)	2.39 (0.66-8.65)
Croatia	M	5.22 (2.58-10.54)	3.93 (2.05-7.54)	**9.41 (4.93-17.04)**	1.42 (0.81-2.49)
	F	7.89 (2.91-21.35)	5.22 (2.70-10.09)	**9.17 (4.17-20.15)**	2.20 (1.14-4.27)
Slovenia	M	**19.28 (6.52-57.02)**	8.11 (4.55-14.47)	6.62 (3.98-11.00)	1.80 (1.06-3.07)
	F	**19.05 (5.18-70.04)**	10.62 (5.83-19.36)	5.04 (2.73-9.32)	2.32 (1.13-4.77)
Germany	M	**10.95 (5.91-20.32)**	8.83 (5.24-14.90)	5.12 (3.24-8.08)	0.92 (0.61-1.40)
	F	**9.32 (4.68-18.58)**	7.10 (4.16-12.13)	4.93 (2.89-8.39)	2.15 (1.38-3.36)
Switzerland	M	6.95 (3.81-12.69)	**9.51 (5.37-16.85)**	7.48 (4.57-12.25)	1.85 (1.15-2.97)
	F	5.13 (2.52-10.45)	**7.76 (4.42-13.60)**	6.85 (3.93-11.94)	2.92 (1.65-5.15)
Bulgaria	M	**10.61 (4.68-24.03)**	4.25 (1.91-9.48)	6.27 (3.10-12.72)	2.97 (1.56-5.68)
	F	**10.48 (3.51-31.27)**	3.91 (1.73-8.81)	6.89 (2.55-18.59)	1.93 (0.89-4.20)
Czech Republic	M	3.20 (1.91-5.36)	**8.39 (4.89-14.39)**	4.95 (3.21-7.62)	2.44 (1.59-3.74)
	F	2.86 (1.63-5.03)	**9.78 (5.73-16.68)**	9.40 (5.78-15.30)	3.48 (2.17-5.50)
Russian Federation (Moscow)	M	4.07 (1.64-10.09)	6.61 (2.22-19.65)	3.12 (1.17-8.30)	3.55 (1.41-8.92)
	F	**13.90 (3.57-54.12)**	4.35 (1.54-12.27)	8.36 (1.59-44.00)	5.57 (1.75-17.71)
Ukraine	M	**5.26 (3.069.04)**	4.40 (2.29-8.48)	3.88 (2.20-6.84)	1.80 (1.01-3.021)
	F	1.78 (0.51-6.20)	**14.32 (4.03-50.82)**	4.77 (1.52-14.94)	4.13 (1.39-12.25)

*M – male; F – female. The highest odds ratios for respective country by sex are in bold. Results are presented as odds ratios and 95% confidence intervals.

Among boys in all countries except Ukraine, perceived marijuana use among older siblings was associated with frequent marijuana use and frequent alcohol use in all countries except Denmark, Estonia, Croatia, and Germany (Table 5 and 6). The strongest associated factor for boys was perceived marijuana availability in four out of eleven countries (Slovenia, Germany, Bulgaria, and Ukraine). For five countries (Denmark, Estonia, Norway, Switzerland, and the Czech Republic), the strongest associated factor was perceived marijuana use among friends.

**Table 6:** Multivariate logistic regression analysis of frequent marijuana use (10+ times) by sex*

Country	Sex	Older siblings smoke marijuana	Parents usually do not know where pupils spend Saturday evenings	Not living with both parents	School performance very good/excellent
Denmark	M	5.82 (3.08-11.00)	3.62 (0.93-14.11)	1.00 (0.52-1.93)	0.78 (0.36-1.65)
	F	5.94 (2.54-13.87)	1.80 (0.16-20.81)	1.75 (0.78-3.92)	3.99 (1.05-15.10)
Estonia	M	5.17 (2.26-11.83)	1.17 (0.48-2.85)	0.91 (0.49-1.69)	1.33 (0.47-3.76)
	F	2.32 (0.61-8.82)	1.96 (0.42-9.19)	0.78 (0.26-2.34)	0.83 (0.15-4.72)
Norway	M	6.03 (2.15-16.93)	0.70 (0.17-2.81)	1.51 (0.57-4.03)	1.17 (0.13-10.48)
	F	3.08 (0.89-10.60)	2.97 (0.60-14.68)	1.27 (0.38-4.25)	3.42 (0.39-30.16)
Croatia	M	4.76 (2.48-9.11)	2.75 (1.26-6.04)	1.42 (0.73-2.78)	1.41 (0.80-2.46)
	F	2.93 (1.43-6.03)	1.79 (0.65-4.96)	1.92 (0.93-3.96)	1.56 (0.82-2.98)
Slovenia	M	3.52 (1.79-6.94)	2.47 (1.12-5.44)	0.62 (0.33-1.17)	1.79 (0.26-12.32)
	F	2.29 (1.18-4.47)	4.05 (1.68-9.79)	1.27 (0.64-2.52)	1.92 (0.40-9.32)
Germany	M	3.30 (2.03-5.38)	1.32 (0.62-2.81)	1.47 (0.95-20.32)	0.97 (0.61-1.55)
	F	2.50 (1.55-4.02)	0.73 (0.28-1.91)	1.80 (1.15-2.82)	1.13 (0.71-1.81)
Switzerland	M	2.17 (1.30-3.65)	1.17 (0.37-3.65)	1.55 (0.91-2.65)	1.13 (0.71-1.80)
	F	2.55 (1.47-4.42)	1.07 (0.24-4.85)	1.13 (0.63-2.06)	1.35 (0.78-2.32)
Bulgaria	M	3.14 (1.29-7.63)	0.91 (0.39-2.12)	0.74 (0.34-1.63)	1.05 (0.47-2.32)
	F	6.13 (2.80-13.41)	1.61 (0.56-4.60)	1.23 (0.52-2.88)	0.71 (0.31-1.58)
Czech Republic	M	3.33 (2.12-5.22)	2.76 (1.10-6.88)	0.75 (0.47-1.21)	1.18 (0.73-1.04)
	F	1.37 (0.84-2.24)	1.81 (0.68-4.85)	1.49 (0.92-2.39)	1.20 (0.74-1.94)
Russian Federation (Moscow)	M	**11.84 (4.26-33.02)**	0.34 (0.09-1.35)	1.53 (0.64-3.68)	0.17 (0.06-0.45)
	F	5.24 (1.84-14.94)	1.40 (0.37-5.27)	0.91 (0.32-2.59)	1.02 (0.18-5.91)
Ukraine	M	1.79 (0.83-3.87)	1.53 (0.72-3.26)	2.06 (1.23-3.46)	1.48 (0.54-4.04)
	F	2.99 (0.95-9.44)	0.85 (0.08-9.05)	2.02 (0.73-5.56)	1.08 (0.29-3.97)

*M – male; F – female. The highest odds ratios for respective country by sex are in bold. Results are presented as odds ratios and 95% confidence intervals.

Among girls, frequent marijuana use was associated with perceived marijuana availability in all countries except Ukraine, perceived marijuana use among friends in all countries except Norway, daily smoking in all countries except in Norway, and frequent alcohol use in all countries except in Norway and Bulgaria. Perceived marijuana use among older siblings was associated with marijuana use in all countries except Estonia, Norway, and Ukraine (Table 6).

Among girls, the strongest associated factor in five countries (Norway, Slovenia, Germany, Bulgaria, and Russian Federation) was perceived marijuana availability; in five countries (Denmark, Estonia, Switzerland, the Czech Republic, and Ukraine), it was perceived marijuana use among friends.

Similarly as for lifetime prevalence of marijuana use, for both sexes in the majority of countries no significant relationship was found between frequent marijuana use and parental control, non-intact family structure, and school performance (Table 6).

Perceived marijuana use among friends was associated with early initiation of marijuana use for boys in all countries except Bulgaria and Ukraine (Table 7). In four countries (Norway, Slovenia, the Czech Republic, and Ukraine), early initiation was associated with perceived marijuana use among siblings. Daily smoking was associated with early initiation for boys in Germany and Switzerland, marijuana availability in Croatia, Russian Federation, and Ukraine, not living with both parents in Croatia, and school achievement in Russian Federation. Frequent alcohol use and parental control were not associated with early initiation of marijuana use among boys (Table 7 and 8).

**Table 7:** Multivariate logistic regression analysis of early initiation of marijuana use by sex*

Country	Sex	Marijuana available “very easy” or “fairly easy”	Most or all friends smoke marijuana	Daily smoking	Frequent alcohol use
Denmark	M	2.67 (0.75-9.48)	**2.56 (1.07-6.12)**	1.29 (0.65-2.57)	0.86 (0.44-1.68)
	F	1.88 (0.39-9.09)	**7.29 (1.77-30.12)**	3.03 (1.13-8.07)	1.86 (0.82-4.22)
Estonia	M	1.09 (0.53-2.21)	**5.96 (2.79-12.71)**	1.64 (0.82-3.30)	1.11 (0.53-2.30)
	F	1.74 (0.53-5.65)	2.25 (0.58-8.83)	**12.04 (1.31-110.46)**	3.30 (1.02-10.69)
Norway	M	0.83 (0.18-3.75)	**54.03 (3.34-875.19)**	3.43 (0.74-15.92)	3.00 (0.64-13.93)
	F	0.40 (0.10-1.62)	4.70 (0.68-32.46)	3.29 (0.55-19.62)	0.59 (0.14-2.42)
Croatia	M	**4.27 (1.39-13.13)**	2.96 (1.38-6.34)	1.10 (0.49-2.47)	2.12 (0.99-4.57)
	F	7.03 (0.88-56.07)	1.50 (0.62-3.59)	1.08 (0.42-2.77)	1.17 (0.49-2.79)
Slovenia	M	1.40 (0.63-3.10)	**2.21 (1.19-4.08)**	1.10 (0.63-1.93)	0.79 (0.45-1.41)
	F	**9.87 (1.30-74.98)**	1.93 (0.99-3.80)	1.18 (0.58-2.39)	1.55 (0.74-3.23)
Germany	M	1.75 (0.91-3.34)	**2.59 (1.57-4.27)**	1.80 (1.09-3.00)	1.05 (0.68-1.63)
	F	1.50 (0.79-2.85)	**2.70 (1.57-4.65)**	1.55 (0.89-2.69)	1.24 (0.79-1.95)
Switzerland	M	1.75 (0.91-3.34)	**2.59 (1.57-4.27)**	1.80 (1.09-3.00)	1.05 (0.68-1.63)
	F	1.05 (0.47-2.31)	1.23 (0.68-2.24)	1.52 (0.84-2.75)	**2.43 (1.35-4.37)**
Bulgaria	M	1.49 (0.52-4.28)	2.43 (0.92-6.44)	1.40 (0.54-3.66)	1.93 (0.81-4.62)
	F	6.44 (0.79-52.41)	1.59 (0.53-4.74)	1.02 (0.26-3.97)	1.71 (0.61-4.77)
Czech Republic	M	1.72 (0.71-4.14)	**2.74 (1.43-5.23)**	1.84 (0.97-3.49)	1.07 (0.5-1.99)
	F	2.47 (0.98-6.21)	**3.34 (1.84-6.04)**	2.03 (1.03-4.00)	2.10 (1.18-3.73)
Russian Federation (Moscow)	M	3.47 (1.08-11.12)	**5.82 (1.79-18.92)**	1.14 (0.32-4.01)	2.09 (0.69-6.37)
	F	0.39 (0.13-1.21)	1.31 (0.37-4.62)	0.76 (0.17-3.38)	3.08 (0.92-10.37)
Ukraine	M	2.34 (1.14-4.79)	1.92 (0.84-4.38)	1.11 (0.52-2.35)	1.33 (0.63-2.81)
	F	1.24 (0.33-4.59)	2.18 (0.59-8.06)	3.90 (0.90-16.84)	4.04 (1.22-13.46)

*M – male; F – female. The highest odds ratios for respective country by sex are in bold. Results are presented as odds ratios and 95% confidence intervals.

**Table 8:** Multivariate logistic regression analysis of early initiation of marijuana use by sex*

Country	Sex	Older siblings smoke marijuana	Parents usually do not know where pupils spend Saturday evenings	Not living with both parents	School performance very good/excellent
Denmark	M	1.48 (0.75-2.93)	4.32 (0.92-20.19)	1.18 (0.59-2.38)	0.44 (0.19-1.01)
	F	1.31 (0.57-3.02)	0.23 (0.01-4.12)	2.80 (1.21-6.49)	3.55 (0.97-12.99)
Estonia	M	1.57 (0.64-3.85)	0.83 (0.30-2.31)	0.75 (0.37-1.50)	0.58 (0.18-1.84)
	F	2.13 (0.52-8.78)	2.77 (0.65-11.82)	0.92 (0.28-3.01)	0.69 (0.07-7.16)
Norway	M	11.47 (3.17-41.48)	0.53 (0.07-4.29)	1.29 (0.33-5.03)	10.37 (0.16-687.21)
	F	0.48 (0.12-1.97)	2.14 (0.43-10.58)	**5.59 (1.47-21.22)**	1.89 (0.30-11.94)
Croatia	M	0.83 (0.36-1.92)	1.76 (0.69-4.53)	2.75 (1.22-6.20)	1.31 (0.63-2.71)
	F	2.09 (0.88-4.99)	0.63 (0.12-3.25)	1.06 (0.40-2.78)	1.11 (0.46-2.65)
Slovenia	M	2.13 (1.09-4.14)	1.88 (0.89-3.96)	0.64 (0.33-1.25)	2.66 (0.29-24.28)
	F	1.57 (0.79-3.09)	0.88 (0.32-2.41)	0.88 (0.43-1.84)	0.36 (0.10-1.30)
Germany	M	1.36 (0.83-2.22)	1.17 (0.56-2.47)	1.42 (0.90-2.26)	0.87 (0.52-1.47)
	F	1.10 (0.69-1.77)	0.88 (0.32-2.40)	1.85 (1.17-2.94)	0.63 (0.39-1.01)
Switzerland	M	1.36 (0.83-2.22)	1.17 (0.56-2.47)	1.42 (0.90-2.26)	0.87 (0.52-1.47)
	F	0.91 (0.51-1.64)	0.77 (0.14-4.20)	1.75 (0.95-3.24)	0.97 (0.55-1.72)
Bulgaria	M	1.98 (0.74-5.31)	1.76 (0.66-4.66)	0.63 (0.21-1.90)	0.94 (0.33-2.70)
	F	1.49 (0.50-4.39)	0.93 (0.17-5.02)	2.25 (0.79-6.39)	0.62 (0.20-1.89)
Czech Republic	M	1.97 (1.07-3.64)	1.73 (0.69-4.38)	1.18 (0.62-2.23)	2.02 (0.85-4.79)
	F	1.21 (0.68-2.17)	1.48 (0.57-3.86)	1.96 (1.10-3.49)	1.13 (0.61-2.08)
Russian Federation (Moscow)	M	2.21 (0.63-7.69)	0.59 (0.14-2.60)	1.05 (0.35-3.13)	0.23 (0.07-0.82)
	F	2.50 (0.75-8.29)	0.60 (0.11-3.37)	0.74 (0.23-2.33)	1.99 (0.21-18.52)
Ukraine	M	**2.74 (1.22-6.16)**	2.14 (0.85-5.37)	1.62 (0.82-3.22)	1.33 (0.28-6.23)
	F	**7.65 (2.18-26.88)**	1.44 (0.10-21.14)	0.54 (0.16-1.85)	0.27 (0.06-1.13)

*M – male; F – female. The highest odds ratios for respective country by sex are in bold. Results are presented as odds ratios and 95% confidence intervals.

The early initiation of marijuana use in girls was not associated with parental control and school achievement in any country. All other factors were scattered, not forming any clear pattern. Daily smoking was associated with early initiation of marijuana use in Denmark, Estonia, and the Czech Republic, frequent alcohol use in Estonia, Switzerland, the Czech Republic, and Ukraine, and marijuana availability only in Slovenia. Perceived marijuana use among siblings was an influencing factor only in Ukraine, and not living with both parents in Denmark, Norway, and Germany.

Perceived marijuana use among friends was the strongest associated factor for early initiation in boys in all countries except Croatia (marijuana availability) and Ukraine (perceived marijuana use among siblings). In Bulgaria, no association with any independent variable was found (Table 7 and 8).

## Discussion

In this study, we investigated the cross-cultural patterns of adolescent marijuana use by eight independent variables describing peer influence, parental control, family structure, and personal risk behavior in 11 countries throughout Europe. We recognized similar influence of cross-cultural contextual factors related to marijuana use regardless of the actual marijuana prevalence in those countries.

The majority of the students in all ESPAD countries who tried any illicit drug used marijuana or hashish. In 2003, the country with the greatest number of students who reported experience with marijuana was the Czech Republic, followed by Switzerland. Low prevalence countries were either found in the south of Europe or among the Nordic countries (11).

Adolescent tobacco use and subsequent cannabis use, according to Mathers et al was not explained convincingly in the summarized studies (14). In Europe, cannabis use is more prevalent among tobacco smokers than among non-smokers (1). In our study, cigarette smoking and marijuana use at least once in lifetime were strongly associated for boys in the majority of countries. It is not surprising because the mode of marijuana use is smoking. Marijuana use at least once in lifetime was also associated with perceived marijuana availability and perceived marijuana use among friends, which is in line with some previous research (15,16). According to our results for girls, perceived marijuana use among friends and marijuana availability were more strongly associated with the lifetime marijuana use than with daily smoking. Influence of frequent alcohol use was in majority of countries (except Norway for the boys and Slovenia and Bulgaria for the girls) associated with lifetime prevalence of marijuana use.

Previous studies showed that high levels of cannabis use were related to poorer educational outcomes, lower income, greater welfare dependence, unemployment, and lower relationship and life satisfaction (5,17). Lower family involvement and increased associations with drug-using peers increase the probability of initiating marijuana use and elevate the frequency of use (16,18). A strong association was found between marijuana use and perceived marijuana availability for both boys and girls, and between marijuana use and perceived marijuana use among friends. According to Kokkevi et al, substance use of older siblings and peers has a strong impact on adolescent marijuana use (12). Our results showed that perceived siblings’ marijuana use was not the strongest associated factor, but contributed to marijuana use.

We found that daily smoking, perceived marijuana availability, and perceived marijuana use among friends and among siblings were the most influencing factors regardless of the prevalence of marijuana use in the respective country.

The association between frequent alcohol use and frequent marijuana use was not so clear, suggesting that alcohol use, although associated to the initiation and experimentation, does not follow the same line of behavior development and that peer habits and environment are at that stage more important.

According to Lynskey et al, association between early cannabis use and later drug use may arise from the effects of the peer and social context within which cannabis is used and obtained (19). In our analyses, early initiation of marijuana use was not strongly associated with the available factors. For boys, it was almost exclusively associated with perceived marijuana use among friends and for girls the pattern was not easy to recognize.

According to our findings, the development of marijuana use behavior in boys and girls follows a different pattern. As for lifetime prevalence among boys (meaning initiation), the associated behavior as daily smoking and alcohol use suggest that the experimentation with marijuana is related to the inclination to personal risk behavior. For girls, even for experimentation, the influence of the group and availability were more important, suggesting that girls might just “follow” what their friends are doing rather than being the initiators or leaders.

Summarized, our results show that marijuana use at least once in lifetime for boys was mostly associated with daily smoking. Frequent marijuana use for both sexes was associated with perceived marijuana availability and perceived marijuana use among friends. Early initiation of marijuana use for boys was associated with perceived marijuana use among friends.

Kokkevi et al found that family-related factors were less important than peer culture (12). Similarly, in line with Sakoman (20) et al that smoking cigarettes, alcohol consumption, and use of drugs are mainly associated with the use of these substances within peer group, our research suggests that the factors most influencing the initiation and continuation of marijuana use originate from young people’s environment. Parental control and family structure may have strong influence until adolescence age, when the strongest influence comes from the environment. Investigating the influence of family, previous studies (21) showed that family cohesion, parental social support, and family interaction (family activities and leisure time spent with family) influenced drug use in adolescents, which is not in accordance with the results of our research. It can be concluded that the quality of family relations, which was a subject of the mentioned study, is more important than the formal family structure and parental control, which was the subject of our study. Although in many aspects parental control, school achievement, and family structure have been strong predictors for risk behavior, including marijuana use, multivariate analysis taking into account available multiple factors in adolescence showed that peer pressure, perceived availability, and other common challenges as drinking and smoking were too strong.

Since a study restricted to one specific sociocultural context has uncertain generalizability, cross-national studies are particularly valuable (12). Our findings may be significant for prevention interventions. Even though countries included into the study belong to various geographical regions (13), our results did not show clear differences among the regions. Moreover, we can conclude that environmental factors have a similar impact on marijuana use regardless of the region. Although cultural differences may play an important role in creation and implementation of the preventive programs, there is enough evidence that they should be multifaceted targeting complex aspects of adolescent life, which are similar regardless of these differences. Dealing with risk behaviors, it is necessary to strengthen an individual’s resistance to the social peer pressure and pay attention to the quality of relationships between adolescents and their peers, parents, and teachers (20). With the support of legal regulations that restrict the availability of dependent substances and family, school environment and peer groups have the strongest influence on adolescent behavior. For prevention and effective treatment interventions, it is very important to understand all personal and environmental factors associated with substance use in adolescence. Environment in which adolescents make their decisions about substance use (school, neighborhood) are ideal places for health promotion. Teachers, parents, and all who live, work, and play with young people should empower them to be able to deal with risks. They should also access all their own resources to be able to help.
